# Language-informed treatment recommendations in psychiatry: bridging the gap in individual predictability

**DOI:** 10.3389/fpsyt.2026.1888061

**Published:** 2026-09-03

**Authors:** Ruyi Shui, Xu Zhang, Zheya Cai, Kangrui Zhao, Michael Siniatchkin, Jiaojiao Hou

**Affiliations:** 1 School of Medicine, Tongji University, Shanghai, China; 2 Department of Child and Adolescent Psychiatry, Psychosomatics and Psychotherapy, Medical Faculty, RWTH Aachen University, Aachen, Germany

**Keywords:** causal inference, individualized treatment effects, machine learning methods, precision psychiatry, treatment recommendation systems

## Abstract

Treatment recommendation systems in psychiatry perform suboptimally, largely due to low individual-level predictability from conventional clinical predictors. We argue that language captured through clinical interviews and electronic health records provides a rich, workflow-native substrate for estimating individualized treatment effects. We highlight opportunities for language-informed systems using modern language models and outline key challenges that must be addressed for safe and scalable deployment.

Mental disorders are a leading and growing cause of disability worldwide, imposing a substantial public-health burden (1). Because etiologies remain only partly understood and patient-level heterogeneity is high, average treatment effects often mask substantial treatment-by-patient interactions; for example, in major depression only ~28–33% remit after first-line therapy (2), and systematic reviews show pronounced heterogeneity of treatment effects across psychiatric interventions (3). Treatment recommendation systems (TRS) use causal machine learning estimators of individualized treatment effects from trials and real-world data to learn treatment rules that maximize expected treatment gain; by selecting the therapy with the highest patient-specific expected benefit, TRS can reduce trial-and-error, shorten time-to-response, improve care efficiency, and provide quantitative benefit–risk information to support shared decision-making (4). Across somatic specialties, TRS-style approaches have seen growing clinical uptake with validated models that inform treatment choice and demonstrate decision utility (5). For example, in inflammatory bowel disease, pre-treatment TRS models can stratify biologic responders and aid initial therapy selection, with multi-center external validations reported (5). By contrast, applications of TRS in mental health remain sparse and their performance inconsistent: models often fail to accurately predict causal response and rarely demonstrate superiority over clinician- or guideline-based choices in decision support, yielding limited incremental clinical utility to date (6).

Individual predictability is the core of TRS (4), providing the common foundation for both causal inference—through the identification and control of bias—and prediction—by enhancing accuracy and decision relevance (4). In psychiatry, individual-level predictability remains low, limiting the effective application of TRS (6). Current, commonly available predictors—such as symptom severity scales, basic demographics, comorbidity, general activity, and simple physiological readouts—provide only limited incremental predictive value for treatment outcomes and often fail to generalize across sites and populations (7). By contrast, novel markers (e.g., electroencephalography- or functional MRI-based neurobiological features, and genetic profiles or polygenic risk scores) can show stronger associations in research cohorts, but clinical use is constrained by acquisition cost, access, and limited real-world validation; where externally tested, accuracy remains moderate and clinical utility unproven (7).

Language—encompassing both speech (e.g., acoustic–prosodic patterns, fluency, coherence) and text (e.g., lexico-semantic/semantic-pragmatic content)—is central to psychiatry because it both constitutes and carries mental symptoms across onset, treatment, and recovery, and has been formalized as a quantifiable clinical signal in major nosologies (e.g., DSM-5 criterion). Recent advances in language models (LMs) have rapidly expanded applications across psychiatry, showing utility beyond diagnosis to decision support in real-world data. For example, Volkmer et al. showed that large language models can analyze and respond to freely written text, making them particularly suitable for processing the large amount of unstructured medical text commonly encountered in psychiatry. By transforming clinical narratives that are otherwise difficult to use directly in machine-learning models into representations suitable for prediction and decision support, LMs may substantially improve the clinical utility of model training (8). In addition, language-based biomarkers can be integrated with cognitive, sensory, and other psychosis-risk markers for multimodal assessment, as language encompasses not only textual content but also speech and other modalities. For instance, Menne et al. treated speech as a potential biomarker and identified robust major depressive disorder (MDD)-related vocal features, including pitch, loudness, and temporal characteristics, suggesting that automated speech analysis may help improve the clinical diagnosis and monitoring of MDD (9). Foltz et al. further clarified that the value of language quantification lies not only in improving classification performance, but also in aligning computational features with specific psychological or clinical constructs. Such construct alignment can enhance model interpretability and generalizability, and may facilitate the incorporation of language features into real-world psychiatric decision-support systems (10). Moreover, the development of Transformer-based architectures has strengthened the ability of LMs to process complex clinical text. A recent study using real-world data from psychiatric inpatients showed that a semi-structured Transformer-based language model outperformed traditional structured-data models across multiple risk-prediction tasks, suggesting favorable stability, robustness, and clinical applicability (11). Unlike costly or restricted markers, language is ubiquitous, low-cost, and naturally longitudinal: they can be produced and captured during routine interviews and documentation, and already reside in clinical notes within the electronic health records (EHRs). Modern clinical natural language processing (NLP) pipelines can slot into existing workflows (e.g., near-real-time texts/speech feeds (12) and EHR-integrated extraction platforms (13)), turning unstructured language into decision-relevant features for risk stratification and decision support. Recent advances in language models have rapidly expanded their applications in psychiatry, extending beyond diagnosis to decision support using real-world data. First, at the level of psychopathology, speech and text capture core symptoms and their fluctuations; reviews and cohort studies show that language features track severity, course, and short-term risk, and outperform traditional baselines in several settings (14). Second, language models and NLP can assist causal inference by extracting information from unstructured notes to measure or proxy confounders and support bias control under potential-outcomes assumptions—thereby moving predictions toward causally grounded, decision-relevant outputs (15). Third, language models enable longitudinal modeling: transformer models integrating clinical notes (and other modalities) learn patient trajectories and forecast near- and longer-term risks in routine care, often improving over conventional statistical models (13). The value of operationalizing and quantifying language lies not only in its potential to improve predictive performance, but also in its capacity to enhance modeling efficiency and clinical interpretability, thereby making models more actionable in real-world clinical settings.

Taken together, we argue that language and modern language models should be incorporated as first-class, supplemental inputs to TRS as they provide rich, low-cost, workflow-native signals for symptom dynamics, support de-biasing via confounder measurement, and model patient-specific trajectories—thereby enhancing individual predictability for treatment selection.

Based on these considerations, we propose a pragmatic pathway for integrating language-informed TRS into psychiatric practice (**Figure 1**).

**Figure 1:**
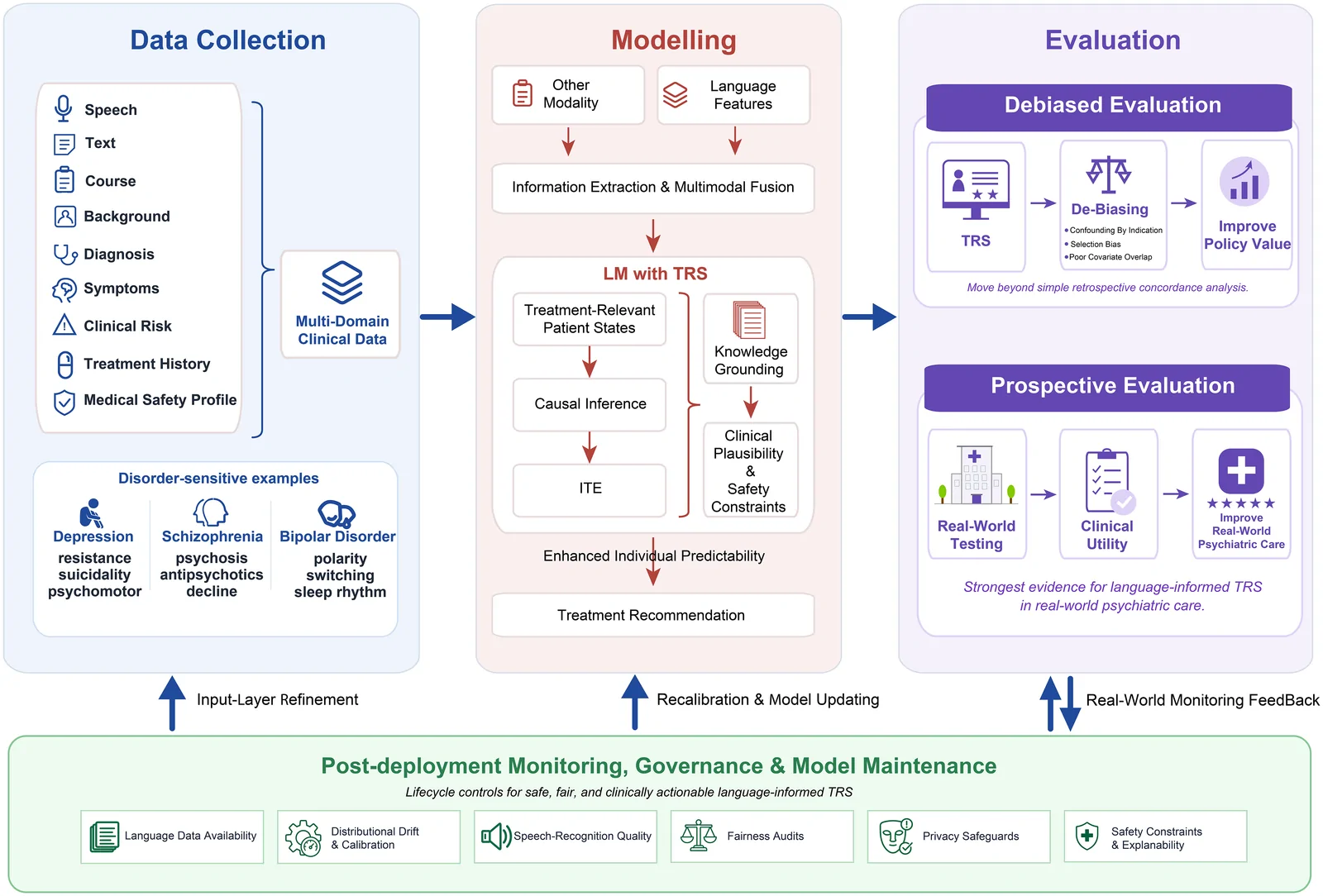
Framework for language-informed treatment recommendation systems in psychiatry. The image shows a workflow in which multimodal clinical data, including speech and text, are integrated into language-informed TRS. Language features and other modalities are fused to construct treatment-relevant patient states, estimate individualized treatment effects, and generate treatment recommendations under knowledge grounding, clinical plausibility, and safety constraints. Evaluation includes debiased retrospective assessment and prospective real-world testing. Post-deployment monitoring supports input refinement, model recalibration, governance, and continuous maintenance. LM, language model; TRS, treatment recommendation system; ITE, individualized treatment effect.

The input layer should be anchored to the treatment decision point. It should use information available before treatment initiation whenever possible (16). Candidate inputs should cover several broad domains: patient background, diagnosis and illness course, baseline symptoms and functioning, clinical risk, previous and current treatment, and medical safety profile (17). These domains are needed because TRS must separate baseline prognosis from treatment-specific benefit (18). Language-derived information should complement these variables. It can capture affective, cognitive, psychotic, suicidal, behavioral, and speech-related phenotypes that are not fully reflected by conventional scales (19). The most relevant inputs will differ by disorder. For major depression, models should pay particular attention to treatment resistance, suicidality, episode course, and psychomotor symptoms (20). For schizophrenia, psychosis dimensions, antipsychotic exposure, treatment resistance, and functional decline may be especially important (21). For bipolar disorder, mood polarity, switching risk, recurrence pattern, and sleep–wake rhythm should be considered (22). Thus, language-informed TRS should not simply add text or speech to existing models. It should use language to build clinically grounded and disorder-sensitive representations of treatment-relevant heterogeneity.

The modeling layer should map heterogeneous inputs onto treatment-relevant patient states (19, 23). LMs can extract clinically meaningful language representations, but these representations should be fused with structured clinical variables to preserve both symptom-level meaning and clinical context. Because psychiatric TRS may influence high-stakes decisions, the model should also include knowledge grounding and safety constraints (23, 24). More concretely, it may be implemented through retrieval-augmented generation (RAG), in which patient-specific cues extracted from interviews, clinical notes, or structured records are used to query a curated and auditable clinical knowledge base, such as psychiatric nosology, symptom taxonomies, treatment guidelines, medication-safety evidence, drug-interaction resources, and local clinical policies (20, 22). This design is consistent with recent mental-health RAG frameworks, in which symptom phrases are first extracted and then used to retrieve guideline-based evidence for subsequent model reasoning. In this setting, the LM extracts and organizes patient information, whereas the retrieval component anchors model reasoning in verifiable clinical evidence. At the modeling level, retrieved evidence can support the interpretation and validation of extracted patient-state representations, provide traceable clinical evidence, constrain the recommendation space, and screen candidate outputs for guideline inconsistency or safety risks (21). These mechanisms complement causal estimation by functioning as an evidence and safety layer around the causal component (15, 22). Before causal estimation, retrieved knowledge may help normalize language-derived phenotypes, map them onto clinically meaningful constructs, and identify contraindications or safety-relevant variables that should be included in the model (22). After causal estimation, the same knowledge layer can check whether the treatment with the highest estimated individualized benefit is guideline-consistent, clinically permissible, and safe for the specific patient. Importantly, safety constraints may create a trade-off with purely causal optimization. A treatment may have the highest estimated individualized treatment effect but still be inappropriate because of contraindications, previous adverse reactions, drug interactions, pregnancy-related concerns, or acute suicide risk. The final recommendation should therefore be interpreted as the treatment with the greatest expected benefit within a clinically permissible and safety-constrained recommendation space, rather than simply the treatment with the highest unconstrained estimated effect (15, 21). It should address treatment selection, confounding and treatment-effect heterogeneity, using methods matched to the estimand, data structure, and clinical decision (4, 15, 16, 25). For language-informed TRS, high-dimensional language embeddings may enter the causal model as covariates, confounder proxies, or treatment-effect modifiers. The choice of causal estimator should depend on the treatment setting, sample size, feature structure, and clinical question. Flexible meta-learners are suitable when heterogeneous treatment effects need to be estimated across candidate treatment strategies using flexible outcome models. Doubly robust learners are preferable when both treatment-assignment and outcome models can be specified with reasonable quality. Causal forests are useful for exploring subgroup heterogeneity and treatment-effect variation in settings with adequate overlap and interpretable covariates. Representation-based neural estimators may be considered when large samples and high-dimensional multimodal embeddings require joint representation learning (23, 24). However, the dimensionality, collinearity, and latent structure of embeddings may weaken positivity and covariate overlap. Practical safeguards include dimensionality reduction or regularization of language representations, mapping embeddings onto clinically interpretable phenotypes, and formal overlap diagnostics. Specifically, overlap should be reported using propensity-score distributions, effective sample size, and representation-space distance or density measures, followed where necessary by trimming patients outside common support and sensitivity analyses for unmeasured confounding (26). In this framework, LMs enhance individual predictability, knowledge grounding improves clinical plausibility and safety, and causal inference translates patient representations into treatment recommendations.

The evaluation layer should move beyond simple retrospective concordance analysis. Many TRS studies compare patients whose actual treatment matched the model recommendation with those whose treatment did not. This approach is intuitive, but it does not provide an unbiased estimate of model-guided benefit (16). In routine care, treatment choice is shaped by illness severity, prognosis, comorbidity, contraindications, patient preference, clinician judgement, and service availability. Therefore, concordant and discordant groups may differ before treatment starts. Their outcome difference may reflect confounding by indication, selection bias, or poor covariate overlap, rather than the effect of following the TRS recommendation (27, 28). Evaluation should therefore be aligned with the causal question. Retrospective studies should report baseline balance and positivity, and should estimate policy value using weighting, matching, or doubly robust methods when appropriate (16, 29). Retrospective studies should report baseline balance and positivity, and should estimate policy value using weighting, matching, or doubly robust methods when appropriate (30, 31). The choice of method should depend on the data structure and clinical question. Propensity-score weighting is useful when measured baseline characteristics differ but covariate overlap is adequate. Matching can provide a transparent comparison among clinically similar patients, although it may exclude patients outside the region of common support. Doubly robust methods are preferable when both treatment-assignment and outcome models can be specified with reasonable quality. For sequential treatment decisions, recurrent outcomes, or time-varying confounding, marginal structural models, g-methods, or dynamic treatment-regime frameworks should be considered (15, 27, 30). Future language-informed TRS studies should meet minimum methodological standards. These include prespecification of the treatment decision point, candidate treatment strategies, target population, follow-up window, outcome definition, and causal contrast (15, 32). They also include reporting of baseline covariate balance for both structured and language-derived variables, assessment of positivity and overlap using propensity-score distributions, effective sample size, and the proportion of patients excluded by matching or trimming, documentation of missingness and availability of language data, and sensitivity analyses for unmeasured confounding (27, 33, 34). Ultimately, the strongest evidence will come from prospective or pragmatic studies that test whether language-informed TRS improves outcomes, safety, and workflow in real-world psychiatric care.

Despite its potential, several important challenges must be acknowledged and addressed. When language features are used as confounder proxies or integrated into treatment effect models, violations of conditional ignorability, positivity, or the stable unit treatment value assumption (SUTVA) (33) can induce substantial bias. Language may encode contextual interactions (e.g., site or interviewer effects) that violate the no-interference component of SUTVA, or act as post-treatment mediators that break consistency. These violations are especially consequential in algorithmic settings, where treatment effect estimators (e.g., meta-learners, doubly robust methods) are highly sensitive to model mis-specification and lack built-in safeguards against structural bias (15, 33). These risks should be addressed through careful temporal alignment of language features with the treatment decision point, site- or interviewer-stratified validation, domain adaptation to reduce site-specific language artefacts, and sensitivity or negative-control analyses to evaluate residual confounding (24). In selected settings, instrumental-variable approaches may also be considered, but only when a clinically credible instrument affects treatment assignment without directly affecting outcomes except through treatment. Second, language features are prone to distributional drift across sites, devices, dialects, and speech recognition systems, and structured EHRs may evolve over time due to workflow or documentation changes, undermining model transportability, calibration, and policy value (35). This issue also raises fairness concerns because automatic speech recognition can show unequal error rates across racial, accent, and dialectal groups, introducing subgroup-specific measurement error before the TRS model is applied (36). Such errors may distort symptom extraction, patient-state representations, and individualized treatment-effect estimates. Studies should therefore report transcription quality across relevant subgroups and assess whether transcription errors change clinically relevant features or treatment rankings (37). Third, language signals encode sociolinguistic variation that can interact with demographic, cultural, and clinical factors, potentially amplifying pre-existing inequities (38). Fairness in language-informed TRS should be defined according to the treatment decision and its expected clinical utility. Calibration across groups is central because predicted benefit and risk should carry comparable clinical meaning across demographic and sociolinguistic groups (39). Equalized odds or equal opportunity can help detect systematic under-recommendation of beneficial treatments or over-recommendation of unsafe treatments, although these criteria may conflict with calibration and overall performance when baseline risks, treatment response distributions, or data quality differ across groups (40). Counterfactual fairness provides an additional check by asking whether a recommendation would change solely because of race, dialect, accent, sex, or cultural-linguistic style after clinically relevant patient states are held fixed (41). Future studies should audit fairness across speech acquisition, transcription, language-feature extraction, causal estimation, treatment ranking, and clinician-facing recommendations, and should report subgroup-specific calibration, error patterns, and policy value, with particular attention to whether biased recommendations could deny effective therapy to disadvantaged groups. Fourth, privacy protection is particularly challenging for language-informed TRS because psychiatric speech and clinical narratives may reveal diagnoses, trauma histories, suicidal thoughts, family dynamics, social context, and rare life events. Simple de-identification is therefore insufficient. A layered strategy is needed, including local processing, federated learning to reduce centralized sharing of raw transcripts or notes, differential privacy for language embeddings or model updates, and clinical-narrative de-identification that covers both direct and contextual identifiers (42–44). Secure data-sharing protocols, including proxy re-encryption approaches, may provide an additional layer of protection for data exchange (45). These methods also have limitations. Federated learning may still leak information through gradients or model updates, differential privacy may reduce model utility or calibration, and de-identification may either miss residual narrative clues or remove treatment-relevant information (42–44). Future systems should report privacy safeguards together with their effects on model performance, fairness, and clinical interpretability. Finally, language-informed TRS often depend on complex language models and causal estimators that are hard to audit or interpret, creating hurdles for clinical trust, transparency, and regulatory acceptance. Explanations should therefore be organized around the treatment decision rather than prediction alone. Patient-level counterfactual explanations can clarify why one treatment is estimated to provide greater benefit than another, but they depend on valid causal assumptions and adequate covariate overlap (46). Feature-attribution methods may identify which structured or language-derived variables contributed to the recommendation, although attribution over high-dimensional embeddings may not map directly onto clinical constructs (10, 47). Evidence-grounded natural-language rationales may improve transparency by linking patient features, estimated treatment benefit, retrieved guideline or literature evidence, and safety constraints, but they should be constrained by evidence retrieval, uncertainty reporting, and clinician review (48).

Future research should move beyond proof-of-concept models toward methodologically robust, clinically translatable frameworks. Clinical translation should also acknowledge that language-informed TRS is not a straightforward plug-in extension of existing workflows. Deployment would require reliable real-time speech-to-text transcription, NLP integration with EHR systems, standardized data capture, regulatory approval, clinician training, patient consent procedures, and ongoing technical support. Beyond initial deployment, language-informed TRS would also require post-deployment monitoring, governance, and model maintenance to ensure safe, fair, and clinically actionable use over time. Together, these steps introduce non-trivial implementation challenges that may affect feasibility, safety, and adoption in routine psychiatric care. First, algorithmic development should prioritize approaches that explicitly encode causal identification assumptions and remain robust when they are partially violated. Second, addressing distributional drift, fairness, and privacy should be embedded upstream—through specialized data governance, workflow standardization, and rigorous, staged validation strategies that reflect real-world variability. Finally, future work should develop auditable, evidence-grounded explanations that make language-informed TRS clinically interpretable, transparent, and actionable, with the strategic use of language as a readily available clinical signal to enable scalable and equitable TRS deployment.

## Data Availability

The original contributions presented in the study are included in the article/supplementary material. Further inquiries can be directed to the corresponding author.
